# N-Acetyltransferase 1 (*NAT1*) Genotype: A Risk Factor for Urinary Bladder Cancer in a Lebanese Population

**DOI:** 10.1155/2012/512976

**Published:** 2012-08-22

**Authors:** Ibrahim A. Yassine, Loulou Kobeissi, Michel E. Jabbour, Hassan R. Dhaini

**Affiliations:** ^1^Faculty of Health Sciences, University of Balamand, Beirut 1100-2807, Lebanon; ^2^School of Public Health, University of California-Los Angeles (UCLA), Los Angeles, CA 90095, USA; ^3^Urology Department, St. George Hospital University Medical Center, Faculty of Medicine, University of Balamand, Beirut 1100-2807, Lebanon; ^4^Medical Laboratory Sciences, Faculty of Health Sciences, University of Balamand, P.O. Box 166378 Ashrafieh, Beirut 1100-2807, Lebanon

## Abstract

In Lebanon, bladder cancer is the second most incident cancer among men. This study investigates a possible association between N-acetyltransferase 1 (*NAT1*) genotype, a drug-metabolizing enzyme coding gene, and bladder cancer in Lebanese men. A case-control study (54 cases and 105 hospital-based controls) was conducted in two major hospitals in Beirut. Cases were randomly selected from patients diagnosed in the period of 2002–2008. Controls were conveniently identified and selected from the same settings. Data was collected using interview questionnaire and blood analysis. *NAT1* genotypes were determined by PCR-RFLP. Statistical analysis revolved around univariate, bivariate, and multivariate logistic regression models, along with checks for effect modification. Results showed *NAT*1^∗^14*A* allele, smoking, occupational exposure to combustion fumes, and prostate-related symptoms, to be risk factors for bladder cancer. The odds of carrying at least one *NAT*1^∗^14*A* allele are 7 times higher in cases compared to controls (OR = 7.86, 95% CI: 1.53–40.39). A gene-environment interaction was identified for *NAT*1^∗^14*A* allele with occupational exposure to combustion fumes. Among carriers of *NAT*1^∗^14*A* allele, the odds of bladder cancer dropped to 2.03 from 3.72. Our study suggests *NAT*1^∗^14*A* allele as a possible biomarker for bladder cancer. Further research is recommended to confirm this association.

## 1. Introduction

Globally, the prevalence of bladder cancer ranks nine and accounts for 3.3% of all malignancies [1]. As a general pattern, incidence is high in industrialized countries such as the United States, Canada, and the European Union, and lower in developing countries in Africa and Asia [2, 3]. In Lebanon, however, bladder cancer incidence increased markedly in the past years [4, 5]. According to the latest Lebanese National Cancer Registry Report, bladder cancer is the second most incident cancer among Lebanese males (ASR = 32 per 100,000) [6].

Geographical and ethnic differences in incidence may be attributed to differences in environmental and genetic factors. Historically, the majority of genetic studies investigating bladder cancer focused on the nuclear genome such as oncogenes, tumor suppressor genes, and subsequent cellular events such as DNA repair, genomic instability, disturbance of gene regulation, alterations of signal transduction, and apoptotic pathways [7, 8]. In the last few years, the role of drug-metabolizing enzymes in bladder cancer has come into focus. Genetic variants of metabolic enzymes are now postulated to result in critical changes in the metabolism of environmental carcinogens and, subsequently, to modify individual bladder cancer susceptibility. Exposure to chemicals, particularly arylamines, through tobacco smoking, fossil fuel emissions, hair dye, and other environmental and occupational sources has been reported as a major risk factor for bladder cancer [9–12]. These suspected carcinogens are normally biotransformed by numerous and complex drug-metabolizing enzymatic pathways. 

Of particular importance are N-acetyltransferase Phase II enzymes known to mediate N-acetylation and O-acetylation reactions of arylamine derivatives. N-acetyltransferase 1 (*NAT1*) gained attention when, contrary to *NAT2*, was shown to be expressed in a wider number of tissues including bladder epithelia [13]. *NAT1* endogenous substances are not well known although there is some evidence that suggests a role for the enzyme in folate metabolism [14]. *NAT1* was originally described as monomorphic based on its selectivity for p-amino-salicylic acid and metabolic yield [15]. The search for a genetic basis to explain differences in NAT1 enzymatic activity led to the discovery of a genetic polymorphism with a reported significant variation in the frequency distribution of the different alleles among different ethnic groups [16–18]. To date, 28 NAT1 allelic variants have been identified and characterized in humans [19].

Inherited differences in acetylation of arylamines may potentially modify individual susceptibility to bladder cancer. Consequently, a population having more of its members carry a “high risk” allele may have a higher bladder cancer risk. The literature reports *NAT*1*14*A* allele to code for a defective enzyme translated into a “slower” acetylation phenotype [20]. Studies also suggest that *NAT*1*10 genetic variant may be associated with elevated levels of NAT1 enzyme activity and was reported to be associated with colon cancer, and to be less frequent among studied groups of bladder cancer patients [18, 21, 22].

The current research was triggered in view of the alarmingly high incidence of bladder cancer in Lebanon, and based on an unusually high *NAT*1*14*A* allelic frequency clustering in a study we previously conducted among a sample of healthy Lebanese-Americans residing in Michigan (*NAT*1*14*A*: 21.4% among Lebanese-Americans compared to 4% or lower among all other populations and ethnic groups investigated for that particular allele) [20, 23, 24]. Our current hospital-based case-control study particularly seeks to investigate the potential association of *NAT1* genetic polymorphism and bladder cancer among Lebanese men residing in Lebanon.

## 2. Materials and Methods

### 2.1. Study Design and Population

**Figure 1:**
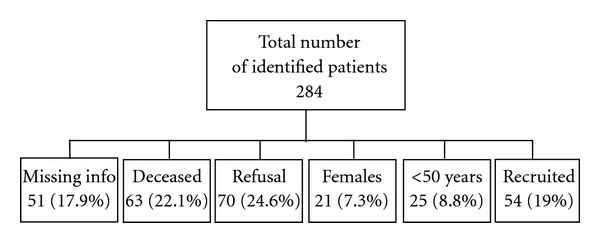
Chart detailing recruitment of bladder cancer cases.

A case-control study was conducted in two major medical centers in the Capital Beirut: St. Georges Hospital and Bahman Hospital. 54 cases and 106 hospital controls were selected. Originally, a total of 284 bladder cancer patients were identified from medical records of both hospitals between 2002 and 2008. Contact information was obtained from the archives of both hospitals. Excluded patients were females, men under age of 50, deceased patients before the beginning of the study, patients with missing contact information, in addition to patients who refused to participate. The remaining patients were included in the study as detailed in Figure 1.

The final sample size was guided by power analysis that revolved around the following: power of 80%, type 1 error of 5%, estimated OR of 3, and a proportion of exposure of *NAT*1*14*A* among cases of 21.4%, based on data observed in earlier studies [20, 23, 24]. A ratio of 1 : 2 of cases to controls was made use of.

#### 2.1.1. Study Inclusion Criteria

Cases were Lebanese males above the age of 50 with a histologically confirmed bladder cancer diagnosed at Saint Georges University Medical Center, and Bahman Hospital between 2002 and 2008. Cases were randomly selected as per year of reporting from the most recent. Controls were conveniently selected from the same settings. These were subjects attending to both settings either for a social visit or a routine checkup. They were Lebanese males, 50 years or older, with no present or previous history of cancer, or any other systemic illness or chronic disease.

#### 2.1.2. Study Exclusion Criteria

The study excluded women, non-Lebanese, and all subjects that are less than 50 years of age for both cases and control groups. In addition, all first-degree relatives were excluded from both cases and control groups. In the control group, all subjects with history of bladder cancer, or other types of cancer or urinary infections, or chronic diseases, were excluded.

### 2.2. Data Collection and Statistical Analysis

University of Balamand and Hospitals IRB approvals were obtained prior to conducting the study. Data collection involved combining structured face-to-face interview questionnaires and blood withdrawal. The questionnaire gathered information on the following: age, family history, smoking habits, alcohol consumption, dietary habits, chronic diseases and urinary infections, use of hair dyes, and occupation. The questionnaire was administered by a trained research assistant in native Arabic. After collection, data were coded, entered, and analyzed using the Statistical Package for Social Sciences (version 16; SPSS, Chicago, IL, USA). Univariate analysis consisted of frequency and percentage distributions for the different categorical variables in the study. Means, SDs, and ranges were computed for the different continuous variables, with checking for normality and outliers. Bivariate analysis mainly used Chi-squared and Fisher's exact test to check for an association between the main outcome variable (urinary bladder) and various exposure and confounding variables. The purpose of this analysis was to examine crude associations and to check for potential confounders and effect modifiers. Multivariate analysis consisted of a backward multivariate logistic regression model, in which analysis included the different exposure and confounding variables that yielded significant results during bivariate analysis. Odds ratios, *P* values, and confidence intervals were computed at a type I error alpha value of 5%. The final model incorporated the exposure and confounding variables that displayed the most significant odds ratios.

### 2.3. DNA Sources and Extraction

2 mL of blood was collected in EDTA-treated tubes from each bladder cancer patient and control subject and stored at 4°C until DNA was extracted. To each blood sample 1 mL of 100% Ficoll was added to improve isolation of buffy coats containing the nucleated white blood cells. This coat appeared after centrifugation at 2500 g for 10 minutes. DNA extraction was performed using a ready-to-use extraction kit (Qiagen) according to manufacturer's instructions and stored at −20°C until genotype analyses.

### 2.4. *NAT1* Amplification and Restriction Digestion

PCR-RFLP assays were used to characterize wild-type and *NAT1* variant alleles as described [25]. Briefly, *NAT1* was amplified by PCR in a 30 *μ*L reaction using 150 ng of genomic DNA as a template, 10 mM Tris-HCL, pH 8.3, 50 mM KCL, 1.5 mM MgCl_2_, 0.2 dNTP, 100 ng of primers NAT1-f (−10 to 29) 5′-TTA GGA ATT CAT GGA CAT TGA AGC ATA TCT TGA AAG AAT-3′ and NAT1-r (1148 to 1127) 5′-GCT TTC TAG CAT AAA TCA CCA A-3′, and 0.75 unit of *Taq* DNA polymerase (Fermentas). The mixture was subjected to a 5 min denaturing at 94°C, followed by 30 cycles of 1 min at 94°C, 1 min at 55°C, 1 min at 72°C, and concluded with a 7 min final extension at 72°C. Following PCR, an aliquot of the product was digested with *Aat*II to confirm amplification. Nucleotide substitution G^350, 351^C (identifier of *NAT*1*5) was detected by digesting *NAT1* PCR product with 10 units of *Ban*I as described above for *Aat*II. Digested samples were run on a 2% agarose gel containing ethidium bromide and examined under UV light.

### 2.5. Nested PCR-Restriction Digestion

**Figure 2:**
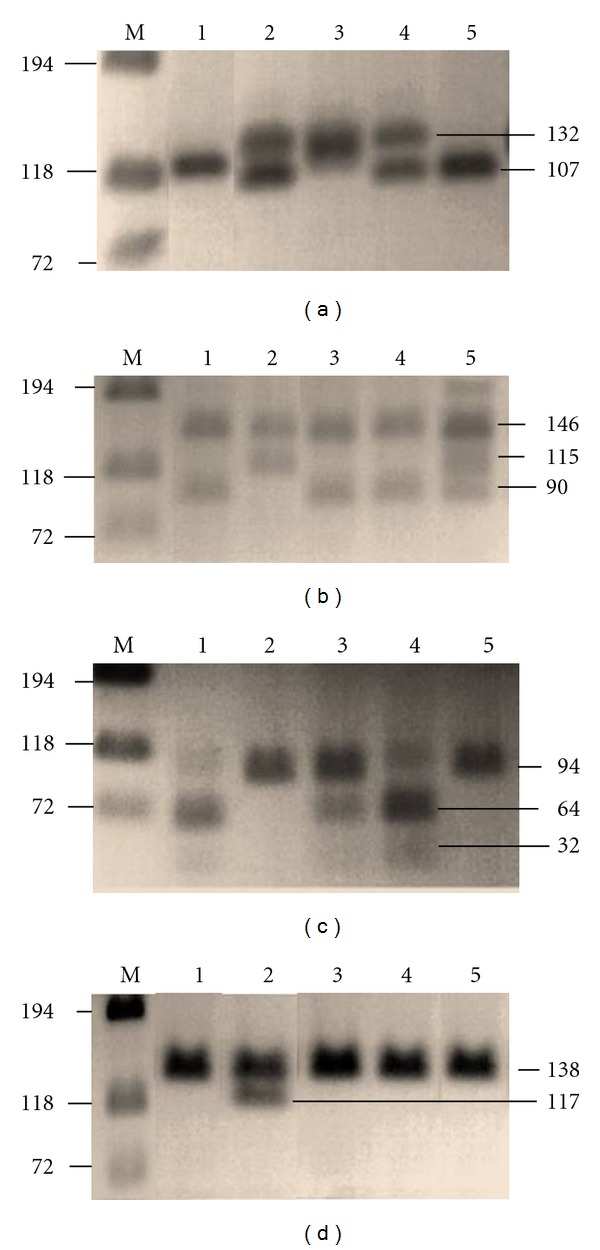
Electrophoresis agarose gel of digested *NAT1* PCR Products. Lane M is the marker phiX174 DNA/BsuRI (Fermentas). (a) *Alw*26I digestion products for C^1095^A detection indicates that Lanes 1 and 5 do not possess the mutation, Lanes 2 and 4 are heterozygous mutants, and Lane 3 is a homozygous mutant. (b) *Hin*fI digestion products for G^560^C detection indicate that Lanes 1, 3, and 4 do not possess the mutation, Lane 5 is heterozygous mutant, and Lane 2 is homozygous mutant. (c) *Ase*I digestion products for T^1088^A detection indicate that Lanes 1 and 4 do not possess the mutation, Lane 3 is heterozygous mutant, and Lanes 2 and 5 are homozygous mutants. (d) *Sca*I digestion products for C^559^T detection indicate that Lanes 1, 3, 4, and 5 do not possess the mutations, while Lane 2 is heterozygous mutant.

For mutations C^1095^A, C^559^T, G^560^C, and T^1088^A, nested PCR was performed. Briefly, a 0.5 *μ*L sample of amplified *NAT1* was used as template in a 20 *μ*L reaction containing 10 mM Tris-HCL, pH 8.3, 50 mM KCl, 1.5 mM MgCl_2_, 0.2 mM each dNTP, 200–350 ng of appropriate primers, and 0.75 units *Taq* DNA polymerase. The mixture was amplified according to conditions shown in Table 1(a). Following PCR, aliquots of the obtained products were digested with restriction enzymes (Fermentas). In order to detect (AAA) after 1091, 15 *μ*L of C^1095^A nested PCR product was digested overnight at 37°C with 2 units of *Mse*I and then was run on a 5% Metaphor gel. All other digested samples were run on a 2% agarose gel containing ethidium bromide and examined under UV light (Figure 2). Genotypes were determined according to consensus nomenclature (Table 1(b)) [19, 26].

## 3. Results and Discussion

### 3.1. Results

Our results show no significant differences between cases and controls in terms of age and education. The average age in cases was 67.1 (±8.1) years compared to 65.6 (±11.3) years in controls. Most subjects had completed junior-high school (around 8 years of education). The average monthly income in both groups was around US $1,800. The majority of cases and controls resided in Beirut (33.3% versus 79%), and Mount Lebanon (46.5% versus 15%) (Table 2). Most of the cases were diagnosed with papillary transitional cell carcinoma (84.9%). Fifty-one percent had a low cancer grade versus 49% with a high grade.

On the other hand, cases were more likely than controls to show clustering of the following alleles: *NAT*1*14*A* (53.7% versus 11.3%, significant), *NAT*1*14*B* (13% versus 4.7%), and *NAT*1*15 (48.1% versus 40.6%). Moreover, cases were less likely than controls to have the clustering of the following alleles: *NAT*1*10 (20.4% versus 50.9%, significant), *NAT*1*3 (13 versus 13.2%), and *NAT*1*4 (25.9% versus 46.2%, significant) (Table 3). *NAT*1*5 and *NAT*1*16 were not detected in any of the cases or controls.

In order to explore potential independent effects and gene-environment interaction, two multivariate models/frameworks were constructed.  Table 4 illustrates results of the multivariate analysis. The best-fit model was constructed at *P* values of 0.1 or lower, both with and without testing for potential gene-environment interaction. The odds of having an income > 1000 US$/month were 7.45 times higher in cases compared to controls, while the odds of an income ranging between 500 and 1000 US$/month were 2.14 times higher in cases than controls. Smoking, prostate-related symptoms, and *NAT*1*14*A* allele were found to be significantly independent risk factors for bladder cancer. Occupational exposure to combustion fumes was also found to be an important risk factor, but not statistically significant. When comparing odds ratios in cases versus controls, the odds of *NAT*1*14*A* allele clustering were 6.94 times higher in cases, while the odds of *NAT*1*10 were 0.74 times (lower in cases compared to controls). In addition, the odds of smoking were found to be 1.02 times higher in cases; the odds of exposure to occupational vapors and fumes were 4.34 times higher in cases; the odds of prostate-related symptoms were 7.81 times higher in cases, compared to controls (Table 4).

When testing for *NAT1* genotype as a potential effect modifier using a best-fit model constructed at *P* values of 0.1 or lower (Tables 4 and 5), results showed the following: cases were 2.17 and 7.12 times more likely to be exposed to occupational combustion fumes in the presence of *NAT*1*14*A* and *NAT*1*10, respectively. However, in the absence of both *NAT*1*14*A* and *NAT*1*10, the odds ratio increased to 12.94.

Similarly, cases were 2.03 times more likely to be exposed to occupational combustion fumes in the presence of *NAT*1*14*A* compared to controls, while, in the absence of *NAT*1*14*A*, cases were 4.47 times more likely to be exposed to occupational combustion fumes compared to controls. In addition, cases were 2.35 times more likely to report prostate-related symptoms in the presence of *NAT*1*14*A*, compared to 1.04 times higher in cases versus controls in the absence of the *NAT*1*14*A* allele.

### 3.2. Discussion

Identification of the underlying causes of bladder cancer in Lebanese men is becoming an urgent need [4, 6, 27–29]. Our study evaluated the effects of *NAT1 *genetic polymorphism on bladder cancer risk. We also investigated a possible interaction of *NAT1* with environmental and occupational risk factors, such as smoking and occupational exposure to combustion fumes. In summary, we found *NAT*1*14*A* to have a high allelic frequency in the total sample (16.5%) and to have a significantly higher clustering among cases compared to controls (OR = 6.9; *P* < 0.05). We also found that the odds of *NAT*1*10 allele carriers are lower among cases compared to controls. In addition, our results present smoking and prostate-related symptoms as independent predisposing factors for bladder cancer.

The hypothesis of *NAT1* affecting bladder cancer risk is not particularly new. Our study, however, reenforces this hypothesis by further investigating the association in a population with both a remarkably high bladder cancer incidence and a reported unusual high frequency of a suspected allele [24]. Our findings confirmed our previous observations in a Lebanese community in Michigan [24]. The reported *NAT*1*14*A* frequency in Lebanese is considered significantly high when compared to other ethnic groups in the literature. For example, *NAT*1*14*A* frequency was reported to be 2.8% in a predominantly Caucasian population [20], 1.3% among a racially diverse population in Los Angeles and Orange Counties of California [30], 1.7% in a population of French Caucasian smokers [31], and 3% in an Australian Caucasian population [23]. Our findings on the association of *NAT*1*14*A* with higher risk, and *NAT*1*10 with lower risk, are consistent with a previously conducted case-control study among a selected German population [32]. 

Although no homozygous *NAT*1*14*A* individuals have been found to date, including the present study, *NAT*1*14*A* has been reported to produce a slow acetylation phenotype based on studies in recombinant enzyme expressed in *E. coli* and blood mononuclear cell lysate [20, 23]. Rodents carrying the slow acetylation phenotype showed a higher DNA adducts formation in the bladder when exposed to aromatic amines [33, 34]. While *NAT2* slow acetylator phenotype is reported to be more prominent [35], *NAT1 *is the main *NAT* expressed in bladder epithelia and cuboidal epithelium of proximal convoluted tubules in the kidney. Recent studies have suggested a novel role for this enzyme in cancer cell growth through folate homeostasis. It is upregulated in several cancer types, and overexpression can lead to increased survival [36, 37]. Many of the effects attributed to *NAT1* and cancer cell growth remain to be explained. Potentially, in a population with higher *NAT*1*14*A* frequency such as this one, *NAT1 *slow acetylation may compete poorly with Phase I bioactivation pathways for arylamines and hence contribute to a higher bladder cancer risk. For *NAT*1*10, on the other hand, reports remain inconsistent. While some studies have reported no change in enzymatic activity for *NAT*1*10, other reports suggest its association with a higher acetylation activity in colon, bladder, and liver tissues [20, 23, 38]. This allele has a point mutation in the 3′-untranslated region thought to alter a poly-adenylation signal, ultimately leading to enhanced mRNA stability. Although our results may suggest a protective role for *NAT*1*10, the low power of the interaction analyses precludes us from making conclusions. Phenotypes may vary with organ/tissue and may be dependent upon other endogenous and environmental factors. Evidence exists for heterogeneity within the “slow” acetylator phenotype [39, 40]. 

 When we tested for interactions, smoking remained to be a significantly independent risk factor. Patients with prostate-related symptoms carrying *NAT*1*14*A* had a higher bladder cancer risk compared to noncarriers. This is an important finding that needs followup using quantitative more accurate measurement tools for prostate-related symptoms assessment. Potentially, partial blockage of bladder outflow, due to a prostate disorder, may increase contact time of chemicals with mucosal cell lining, thereby increasing odds of malignant transformation, particularly in carriers of the slow *NAT*1*14*A* phenotype. On the other hand, our analysis suggests a lower risk for *NAT*1*14*A* carriers exposed to occupational combustion fumes compared to noncarriers. This needs further investigation before drawing conclusions. It may relate, in part, to differences in chemical exposure, other genetic factors related to metabolism, or potentially the small sample size. Our study did not investigate schistosomiasis as a potential risk factor since this infection is very rare in Lebanon and may be more relevant in countries like Egypt [41]. Schistosomiasis is known to be associated with squamous cell carcinoma (SCC) [42]. The majority of our cases had papillary transitional cell carcinoma and absence of SCC.

Several limitations could have affected the results of the current study. The external validity of this case-control study is limited due to the selection of a hospital-based sample that might overshadow the ability to generalize to the overall population. A larger sample size is also needed for increasing the power of this study to be able to demonstrate a higher confidence when interpreting the observed results of effect modifiers. Although our sample size was guided by power analysis, it would have been more optimal to include larger number of cases (double or triple than the minimum sample size calculated). This was not possible due to the high proportion of refusals, deceased, and subjects with no contact information (added together, they make 65% of the total number of identified patients). Another limitation of the study is the possibility of other genetic polymorphisms, such as *NAT2*, that may modify bladder cancer risk, in addition to other risk factors not investigated by the current study. This is mainly due to limitations in both sample size and budget. Further studies should be conducted in the future to address these points. An important limitation worth noting is related to a possible misclassification of the *NAT*1*4-wild type allele. *NAT*1*4 may include other mutations not tested for in the current study. This may bias our risk estimates toward the null. However, many of these nucleotide substitutions and corresponding *NAT1* alleles are reported to be very rare and reflect nucleotide diversity (frequency of 0.003–0.005) rather than genetic polymorphism, with many of them silent [43]. At the same time, our investigation targeted particular alleles based on results we obtained previously from the Michigan group.

On the other hand, several strengths should also be highlighted in this case-control study. The quality of the measures used is high, particularly due to combining two formats of data collection: interview questionnaire and genetic predisposition analysis. No major challenges existed for adjusting for missing and non-response data. In addition, several multivariate models were analyzed before determining the best-fit models used. All the different constructed models yielded similar patterns in the observed results, which reflect the high internal validity of the observed findings reported herein. 

## 4. Conclusions

In conclusion, this is the first case-control study in Lebanon attempting to explain the high incidence of bladder cancer. Our results suggest that *NAT*1*14*A* influences bladder cancer risk in Lebanese males. Although the observed effects may be moderate, the suggested association between incidence and genetic variation may account for a substantial proportion of cases in the Lebanese context. On one hand, the *NAT1* enzyme was recently reported as a biomarker of cancer, and its overexpression was found to be associated with increased resistance to chemotherapy [36]. This finding may have important prognostic impact in predicting clinical outcomes among bladder cancer patients. *NAT1 14A* may be a viable candidate for preclinical and clinical evaluation, as well as for drug development. On the other hand, early screening and detection of *NAT*1*14 in high-risk groups, such as smokers, may be important for Lebanese in view of the double burden of the two highest cancer incidents: lung and bladder. We recommend building upon these observations by carrying out larger multicenter studies incorporating larger sample sizes. Although individual risks associated with N-acetylation genotypes are small, they do increase when considered in conjunction with other metabolizing-enzyme genes, susceptibility genes, and chemical exposures. Because of the relatively high frequency of *NAT*1*14*A* in the Lebanese population, the attributable risk of bladder cancer in this population may be high. However, future studies must be directed toward investigating the contribution of the combined effect of other drug-metabolizing enzymes, particularly *NAT2* acetylator polymorphism. 

## Figures and Tables

**Table:** (a) PCR-RFLP primers, enzymes, and conditions for *NAT1* genotyping

Detected mutation		Annealing T°C	Expected	Restriction
	Primers		fragment	digestion at
			size in bp	37°C
C^1095 ^A	1095-F 5′-CAT TTC ACC TAT AAA AAT GTC-3′	58	132	*Alw*26I
	1095-r 5′-CCA AGA TAA CCA CAG GCC ATC TTT AAG A-3′			
				
C^559 ^T	Mt2-f 5′-ATC TCC TAG AAG ACA GCA AGT AC-3′	55	138	*Sca*I
	Mt2-r 5′-AGG AAC AAA ATG ATT TAC TA-3′			
				
G^560^ C	SJ-f 5′-CTC CTG CAG GTG ACC ATT GAT-3′	55	261	*Hin*fI
	SJ-r 5′-CTT AAG AGT AAA GGA GTA GAT GAT T-3′			
				
	1088-f 5′-CAA ACC TTT TCA AAT AAT AAT AAT AAT AAT			
T^1088^ A	ATT AA-3′	55	96	*Ase*I
	1088-r 5′-GCT TTC TAG CAT AAA TCA CCA A-3′			

**Table:** (b) * NAT1* haplotypes consensus nomenclature [19]

*NAT1* haplotypes	Nucleotide change(s) and rs identifier(s)
NAT1*4	Reference gene sequence published in GenBank:
	accession number X17059
	
NAT1*3	1095C > A (rs15561)
	
NAT1*5	350,351G > C (rs72554606)
	497–499G > C (rs72554608)
	884A > G (rs55793712)
	Δ^976^ (rs72554612)
	Δ^1105^ (rs72554613)
	
NAT1*10	1088T > A (rs1057126)
	1095C > A (rs15561)
	
NAT1*14A	560G > A (rs4986782)
	1088T > A (rs1057126)
	1095C > A (rs15561)
	
NAT1*14B	560G > A (rs4986782)
	
NAT1*15	559C > T (rs5030839)
	
NAT1*16	[AAA] immediately after 1091
	1095C > A (rs15561)

**Table 2:** Sociodemographic background of cases and controls (*n* = 160).

Variables		Bladder cancer cases	Control
Age	Mean (SD)	67.1 (8.1)	65.6 (11.3)
		*P*-value = 0.39	

Years of education	Mean (SD)	8.26 ± 6.76	7.88 ± 5.34
		*P*-value = 0.69	

Salary (US$)	Mean (SD)	1721 (2435)	1885 (2230)
		*P*-value = 0.003	

Salary (US $)		*N* (%)	*N* (%)
Less than 500 $		11 (27.5)	36 (52.9)
Between 500 $ and 1000 $		16 (40)	27 (39.7)
Between 1000 $ and 5000 $		12 (30)	5 (7.4)
More than 5000 $		1 (2.5)	0 (0)

Current residence			
Beirut		18 (33.3)	79 (79)
Mount lebanon		25 (46.3)	15 (15)
North		2 (3.7)	4 (4)
South		4 (7.4)	2 (2)
Bekaa		5 (9.3)	0 (0)
		*P*-Value = 0.00	

Prostate-related symptoms			
Yes		28 (51.9)	23 (21.9)
No		26 (48.1)	82 (78.1)
		*P*-Value = 0.00	

Suffering from UTI problems			
Yes		2 (3.7)	23 (21.9)
No		52 (96.3)	82 (78.1)
		*P*-Value = 0.03	

Histological type and grade		Bladder Cancer Cases* N* (%)	
Papillary transitional cell carcinoma		45 (84.9)	
Transitional cell carcinoma (NOS)		5 (9.4)	
Adenocarcinoma (NOS)		1 (1.9)	
Unspecified bladder cancer		2 (3.8)	
Low grade		26 (51)	
High grade		25 (49)	

**Table 3:** Bivariate analysis on *NAT1* alleles clustering among cases and controls (*n* = 160).

	Cases	Controls
Allele clustering	*N* (%)	*N* (%)
NAT1*15	26 (48.1)	43 (40.6)
	*P* value = 0.36	
NAT1*14B	7 (13)	5 (4.7)
	*P* value = 0.06	
NAT1*14A	29 (53.7)	12 (11.3)
	*P* value = 0.00	
NAT1*10	11 (20.4)	54 (50.9)
	*P* value = 0.00	
NAT1*3	7 (13)	14 (13.2)
	*P* value = 0.96	
NAT1*4	14 (25.9)	49 (46.2)
	*P* value = 0.013	
Familial history of cancer		

Yes	18 (33.3)	8 (7.6)
No	36 (66.7)	97 (92.4)
	*P* value = 0.00	

**Table 4:** Multivariate logistic regression analysis testing for independent effects of suspected risk factors, as well as for a potential gene-environment interaction.  The used best-fit model is based on the following variables in the crude model: income, family history, passive smoking at the work place, occupational exposure to combustion fumes, previous history of prostate-related symptoms, and history of urinary tract infections.

Variable	Testing for independent effect	Testing for potential gene-environment interaction		
		NAT1 *14A	NAT1 *10	
	Adjusted OR (CI)	Adjusted OR (CI)	Adjusted OR(CI)	Noneadjusted OR (CI)
Income (reference < 500 $/month)				
500–1000	2.14 (0.4–11.4)	1.06 (0.05–21.2)	5.87 (0.26–133.2)	14.59 (0.04–5318.5)
>1000	7.45 (0.91–61.2)	2.18 (0.04–114.1)	10.81 (0.085–1369.54)	11.45 (0.098–1334.9)

Smoking	1.02* (1.01–1.04)	1.024* (0.991–1.06)	1.017 (0.99–1.04)	1.039 (0.98–1.104)

Occupational exposure to combustion fumes (reference none)	4.34 (0.83–22.6)	2.17 (0.07–70.33)	7.12 (0.28–182.8)	12.94 (0.00−)

Prostate-related symptoms (reference: none)	7.81* (1.14–53.41)	2.94 (0.00−)	3.04 (0.074–125.1)	

Allele (reference: none)				
NAT1 *14A	6.937* (1.19–4.04)			
NAT1 *10	0.741 (0.099–5.54)			
*NagelKerke R* ^2^	0.597	0.64	0.442	0.46

*Significant at or less than 0.05.

**Table 5:** Multivariate logistic regression analysis testing for NAT1 *14A as a potential effect modifier.  The used best-fit model is based on the following variables in the crude model: income, family history, passive smoking at the work place, occupational exposure to combustion fumes, previous history of prostate-related symptoms, and history of urinary tract infections.

Variable	Testing for independent effect	Testing for NAT1 *14 as a potential effect modifier	
		NAT1 *14A (yes)	NAT1 *14A (no)
	Adjusted OR (CI)	Adjusted OR (CI)	Adjusted OR (CI)
Income (reference < 500 $/month)			
500–1000	2.12 (0.41–11.04)	0.58 (0.015–22.29)	4.82 (0.47–49.9)
>1000	6.2 (0.77–50.04	1.39 (0.013–145.7)	7.26 (0.38–137.9)

Smoking	1.02* (1.00–1.037)	1.02 (0.98–1.06)	1.02* (1.00–1.043)
Occupational exposure to combustion fumes (reference none)	3.72 (0.74–18.8)	2.03 (0.054–75.45)	4.47 (0.47–42.25)
Prostate-related symptoms (reference: none)	6.42* (0.92–39.1)	2.35 (0.00−)	1.04 (0.039–28.12)
Allele (reference: none)			
NAT1 *14A	7.86* (1.53–40.39)		
*NagelKerke R* ^2^	0.57	0.62	0.47

*Significant at or less than 0.05.
